# Drug-Induced Interstitial Lung Disease: A Systematic Review

**DOI:** 10.3390/jcm7100356

**Published:** 2018-10-15

**Authors:** Sarah Skeoch, Nicholas Weatherley, Andrew J. Swift, Alexander Oldroyd, Christopher Johns, Conal Hayton, Alessandro Giollo, James M. Wild, John C. Waterton, Maya Buch, Kim Linton, Ian N. Bruce, Colm Leonard, Stephen Bianchi, Nazia Chaudhuri

**Affiliations:** 1 Arthritis Research UK Centre for Epidemiology, Division of Musculoskeletal and Dermatological Sciences, School of Biological Sciences, Faculty of Biology Medicine & Health, University of Manchester, Manchester Academic Health Sciences Centre, Manchester M13 9PL, UK; sarah.skeoch@manchester.ac.uk (S.S.); Alexander.Oldroyd@manchester.ac.uk (A.O.); Ian.Bruce@manchester.ac.uk (I.N.B.); 2 Royal National Hospital for Rheumatic Diseases, Royal United Hospitals Bath NHS Foundation Trust, Bath BA1 1RL, UK; 3 Department of Infection, Immunity & Cardiovascular Disease, University of Sheffield, Sheffield S10 2TN, UK; nickweatherley@doctors.org.uk (N.W.); A.J.Swift@sheffield.ac.uk (A.J.S.); c.johns@sheffield.ac.uk (C.J.); J.M.Wild@sheffield.ac.uk (J.M.W.); 4 North West Lung Centre, Manchester University NHS Foundation Trust, Manchester Academic Health Science Centre, Manchester M6 8HD, UK; conalhayton@doctors.org.uk (C.H.); Colm.Leonard@nice.org (C.L.); 5 Leeds Institute of Rheumatic and Musculoskeletal Medicine, NIHR Leeds Biomedical Research Centre, University of Leeds, Leeds LS2 9JT, UK; A.Giollo@leeds.ac.uk (A.G.); M.Buch@leeds.ac.uk (M.B.); 6 Rheumatology Unit, Department of Medicine, University of Verona, 37134 Verona, Italy; 7 Bioxydyn Limited, Rutherford House, Manchester Science Park, Manchester M15 6SZ, UK; john.waterton@manchester.ac.uk; 8 Centre for Imaging Sciences, Division of Informatics Imaging & Data Sciences, School of Health Sciences, Faculty of Biology Medicine & Health, University of Manchester, Manchester Academic Health Sciences Centre, Manchester M13 9PL, UK; 9 Division of Cancer Sciences, School of Medical Sciences, Faculty of Biology Medicine and Health, University of Manchester, Manchester Academic Health Sciences Centre, Manchester M13 9PL, UK; Kim.Linton@manchester.ac.uk; 10 The Kellgren Centre for Rheumatology, NIHR Manchester Biomedical Research Centre, Manchester University NHS Foundation Trust, Manchester Academic Health Science Centre, Manchester M6 8HD, UK; 11 Academic Directorate of Respiratory Medicine, Sheffield Teaching Hospitals NHS Foundation Trust, Sheffield S10 2JF, UK; Stephen.Bianchi@sth.nhs.uk

**Keywords:** drug-induced interstitial lung disease, pulmonary toxicity, drug-induced pneumonitis

## Abstract

Background: Drug-induced interstitial lung disease (DIILD) occurs as a result of numerous agents, but the risk often only becomes apparent after the marketing authorisation of such agents. Methods: In this PRISMA-compliant systematic review, we aimed to evaluate and synthesise the current literature on DIILD. Results: Following a quality assessment, 156 full-text papers describing more than 6000 DIILD cases were included in the review. However, the majority of the papers were of low or very low quality in relation to the review question (78%). Thus, it was not possible to perform a meta-analysis, and descriptive review was undertaken instead. DIILD incidence rates varied between 4.1 and 12.4 cases/million/year. DIILD accounted for 3–5% of prevalent ILD cases. Cancer drugs, followed by rheumatology drugs, amiodarone and antibiotics, were the most common causes of DIILD. The radiopathological phenotype of DIILD varied between and within agents, and no typical radiological pattern specific to DIILD was identified. Mortality rates of over 50% were reported in some studies. Severity at presentation was the most reliable predictor of mortality. Glucocorticoids (GCs) were commonly used to treat DIILD, but no prospective studies examined their effect on outcome. Conclusions: Overall high-quality evidence in DIILD is lacking, and the current review will inform larger prospective studies to investigate the diagnosis and management of DIILD.

## 1. Introduction

Drug-induced interstitial lung disease (DIILD) occurs when exposure to a drug causes inflammation and eventually fibrosis of the lung interstitium. Over 350 drugs may cause DIILD, but liability is often not recognised until late in drug development, or after launch. New causative drugs are regularly identified, with over 1300 drugs, procedures or substances reported to cause respiratory problems on the comprehensive Pneumotox website (www.pneumotox.com). DIILD is a recognised subtype of diffuse parenchymal lung diseases according to the American Thoracic Society/European Respiratory Society (ATS/ERS) classification [1], but clinical, pathological and radiological features are rarely specific and difficult to distinguish from other interstitial pneumonias. Moreover, the clinical phenotype, imaging and histopathology patterns vary significantly between drugs and between patients on the same drug. DIILD is consequently a diagnosis of exclusion, and this poses unique challenges for the treating physician and for the study of DIILD in both epidemiological and drug development settings.

DIILD is diagnosed on the basis of clinical, physiological and radiological findings consistent with ILD; a temporal relationship between onset of symptoms and drug exposure; absence of another more likely cause, e.g., infection, pulmonary oedema, radiation-induced lung injury, progression of the underlying disease; and improvement upon withdrawal of the suspected causative agent with or without corticosteroid therapy and, in some cases, deterioration upon re-challenge. An internationally agreed severity classification is used in clinical trials (Table 1) [2].

Drugs and patterns of disease are catalogued in the International Database “Pneumotox” (www.pneumotox.com). However, there is no detailed overall picture of the incidence, phenotype and clinical course of DIILD. The Translational Imaging in Drug Safety Initiative (TRISTAN) consortium is designing prospective studies to develop sensitive and specific biomarkers in patients with DIILD (http://www.imi-tristan.eu). The aim of this systematic review is to summarise the current knowledge of DIILD as a useful guide to clinicians and to inform the TRISTAN studies.

## 2. Methods

**Figure 1:**
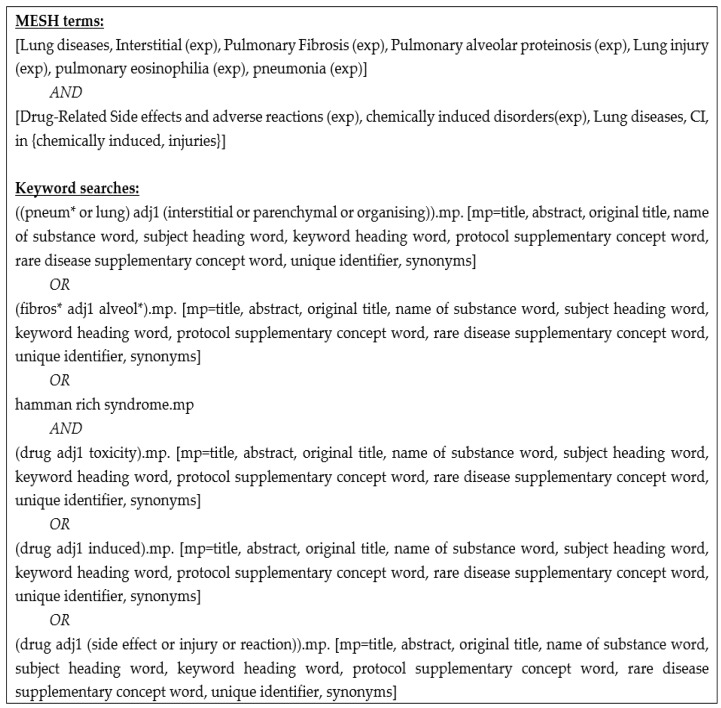
Search terms used in Medline, Embase and Cochrane Register of Controlled Trials.

We conducted a systematic review of observational studies in accordance with the Preferred Reporting Items for Systematic Reviews and Meta-Analyses consensus guidelines (PRISMA) [3], with the aims of: (1) determining the incidence and prevalence of DIILD, (2) identifying common causative drugs, (3) identifying risk factors for DIILD, (4) comparing imaging and non-imaging investigations for assessment and diagnosis of DIILD, (5) assessing the prevalence of DIILD subtypes, (6) measuring the impact of glucocorticoid therapy on outcomes and (7) defining the prognosis of DIILD. We searched Medline, Embase and The Cochrane Register of Controlled Trials in May 2017 using the Medical Subject Headings (MESH) and keyword searches detailed in Figure 1. The following exclusion criteria were applied: studies not in English language, non-human studies, sample size of less than 10, DIILD related to non-licensed drugs and chemicals, e.g., alcohol or organophosphates. Two authors independently screened the titles and abstracts for eligibility. In circumstances where it was not clear from the abstract that the study was eligible, the paper was included in the full-text review. Any disagreements regarding abstract inclusion were resolved by a third independent reviewer. Additional papers were also identified through grey (manual) searches. Full texts of eligible papers were obtained and assessed for inclusion in duplicate, with two reviewers allocated to each question. In the case of overlap between articles reporting the same cohort, we included the study with the largest cohort. The quality of evidence and risk of bias were assessed using the Grading Recommendations Assessment and Development Evidence (GRADE) criteria with supporting guidance from the Cochrane website [4]. Data were extracted using the Population, Intervention, Comparison, Outcome (PICO) framework and included title, year of publication, study design, sample size, study population, patient characteristics, intervention and comparator (where applicable), outcomes, funding and conclusions. The study was prospectively registered on the PROSPERO website (registration number: CRD42017071276). Data were evaluated for inclusion in a meta-analysis based on quality and bias, and, if excluded, descriptive synthesis was undertaken.

## 3. Results

**Figure 2:**
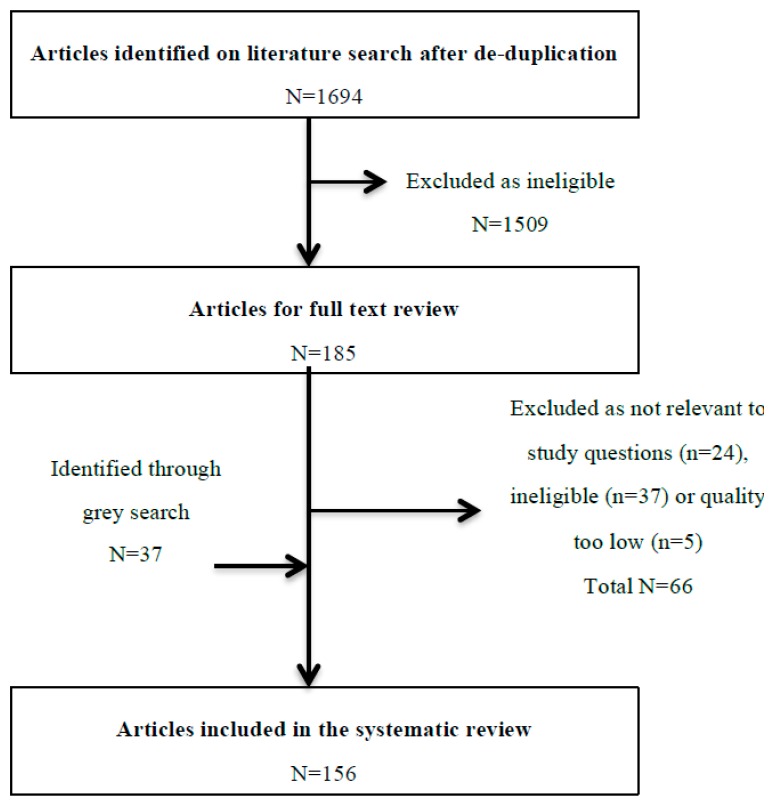
Flow diagram of the review process from abstract review to final inclusion. In total, these 156 articles report approximately 6200 patients with confirmed or suspected DIILD, which was fatal in around 672/2647 (25.4%) cases.

**Figure 3:**
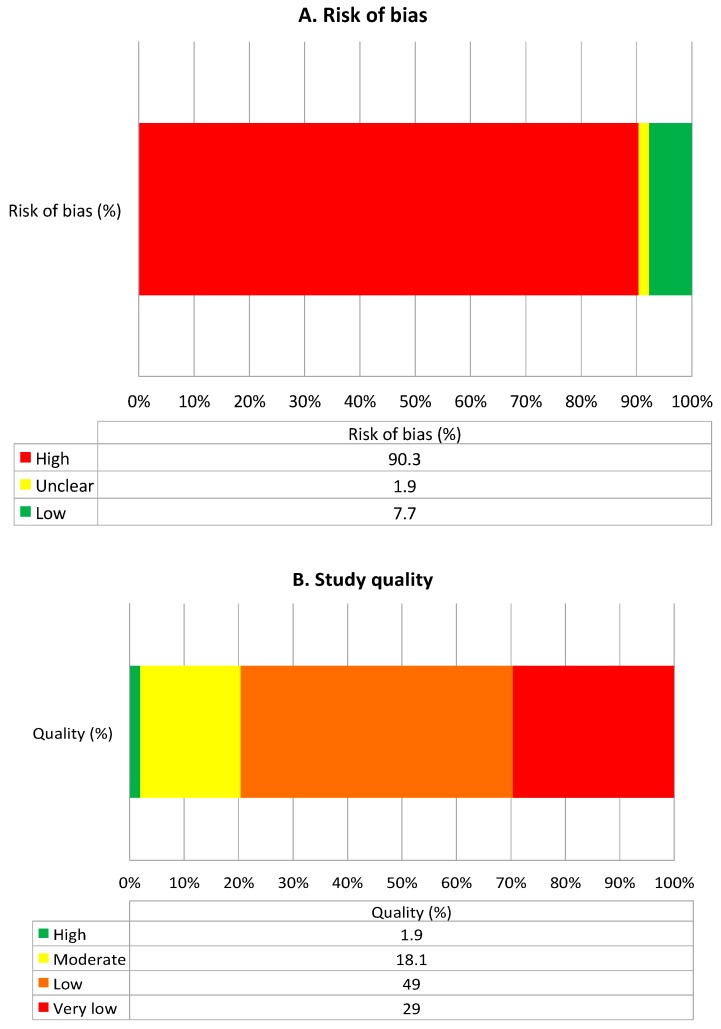
Summary of quality and bias, as assessed using the Grading Recommendations Assessment and Development Evidence (GRADE) method [4]. (**A**) Summarises risk of bias and (**B**) summarises quality of included studies.

After de-duplication, we reviewed 1694 titles and abstracts; from these, we included 185 (10.9%) in a full-text review (Figure 2). A further 66 articles were excluded after full review, and 37 were included in grey searches, providing a total of 156 articles eligible for inclusion. The most common reasons for exclusion of full-text papers were that the study provided no information relevant to the study questions (e.g., described all adverse drug events without specific information on DIILD) (*n* = 24), it took the form of a narrative review (*n* = 20) or the sample size was less than 10 (*n* = 12). Of those where sample size was less than 10, only one drug was not described in the other included papers (hydroxyurea). Potential biases and the quality of included articles are described in Figure 3. The majority were low or very low quality (78%), and 90% had a high risk of bias, mainly due to significant limitations in design, poor precision and indirectness of the study population. Another major limitation was the lack of standardised case definition for DIILD; some studies used physician-reported diagnosis, others used radiological evidence of ILD to define cases without an assessment of clinical characteristics or exclusion of infection, and workup to exclude another competing diagnosis was not stated, minimal or absent. In many studies, a significant proportion of subjects were asymptomatic. A number of post-marketing studies attempted to address this with an expert panel case review and verification process. However, the overall lack of standardised case definition and a paucity of independent case verification hampered any quantitative data synthesis.

We also noted a geographical bias, with more than one-third of the studies (mainly large post-marketing registers) being reported from Japan. A higher prevalence of reported ILD compared to the West has previously been noted in Japanese populations; however, much of this has been suggested to be artefactual due to coding and spontaneous reporting practices, rather than biological reasons [5]. The Proportional Reporting Ratio method was not used [6].

Due to the overall poor quality of evidence, meta-analyses for individual questions were not possible, and a descriptive review was subsequently undertaken.

### 3.1. Incidence and Prevalence

The reported incidence of DIILD for individual drugs ranged from <1% to almost 60% (Table 2). Many studies relied on spontaneous physician or administrative reporting and are susceptible to reporting bias for positive cases.

At a population level, a study from the Clinical Practice Research Database (UK) between 1997 and 2008 reported an incidence of 4.1 per million per annum [7] based on 128 patients with drug- (*n* = 22) or radiation-induced (*n* = 106) ILD. This is likely to be a significant underestimate, considering the case definitions they used and the primary care nature of the cohort. Furthermore, since these figures were published, there has been a significant expansion of new oncology drugs with a high rate of DIILD (www.pneumotox.com). A more recent study in a county within Greater Paris estimated the incidence of all ILD cases at 19.4 per 100,000 per year based on both primary and secondary care data [8]. DIILD accounted for 6.4% of incident cases, suggesting a rate of 12.4 cases per million per year. However, the authors acknowledged that the population was not representative of the demographic structure of the French general population. A few studies also examined rates of DIILD within ILD populations [8,9,10,11]. Four ILD cohorts of 848, 237, 460 and 431 cases of ILD estimated the prevalence of DIILD to be 3%, 3%, 2.6% and 5%, respectively [8,9,10,11].

### 3.2. Common Causative Drugs

We identified six single-centre studies that comprehensively reported DIILD rates for individual drugs in unselected DIILD cohorts [12,13,14,15,16,17]. Not all studies reported DIILD as the primary objective: five were retrospective studies and four were from a single region (Japan). Cancer drugs were the leading cause of DIILD in most studies, accounting for 23–51% of cases, followed by disease-modifying anti-rheumatic drugs (DMARDs) (6–72%), antibiotics (6–26%), non-steroidal anti-inflammatory agents (NSAIDs) (0–23%), psychiatric medications (0–9%) and anti-arrhythmic agents (0–9%). In the Flanders ILD registry, amiodarone followed by nitrofurantoin were the most common causes [10]. Table 1 summarises the incidence and mortality rates for DIILD studies relating to specific agents or drug classes. Consistent with results from unselected DIILD cohort studies, cancer drugs accounted for the majority of drug-specific published studies identified in the initial search (*n* = 37), followed by DMARDs (*n* = 12), cardiology medications (*n* = 10) and antibiotics (*n* = 7).

#### 3.2.1. Cancer Therapy

Identifying specific causative agents is challenging in oncology when drugs are given in combination regimens, or in association with thoracic radiotherapy, which is independently associated with lung fibrosis. The most common individual cancer drugs causing DIILD were identified as bleomycin, gemcitabine, epidermal growth factor receptor (EGFR)-directed therapies, mechanistic target of rapamycin protein (MTOR) inhibitors and immune checkpoint inhibitors. Methotrexate, which is used for the treatment of cancer as well as rheumatological conditions, was also identified.

##### Bleomycin

Bleomycin, used predominantly to treat Hodgkin’s lymphoma and germ cell tumours, causes lung injury via immune-mediated and direct toxic effects [18,19]. The reported risk is 6.8–21%, with an associated mortality rate of up to 48% [18,19,20,21,22,23]. The clinical presentation of bleomycin lung injury is highly variable but can be asymptomatic. Up to 39% of cases are detected on imaging alone [22,23]. Pulmonary physiology changes are common and include an early reduction in diffusing capacity of the lung for carbon monoxide (D_LCO_) followed by changes in forced vital capacity (FVC), which correlates with symptomatic deterioration [18,22].

DIILD can occur at any time during treatment [18]. A study in germ cell tumour patients treated with high-dose bleomycin reported a median time from bleomycin initiation to DIILD of 4.2 months [23]. In this study, cumulative doses >300,000 international units were associated with a 3.5-fold increased risk of DIILD. Idiosyncratic reactions at low doses early in the treatment course are also less commonly described [18,23]. Recent advances in positron emission tomography-directed omission of bleomycin in selected patients with Hodgkin’s lymphoma have been associated with a significant reduction in pulmonary toxicity [24].

##### Gemcitabine

Gemcitabine is used to treat a range of cancers, including non-small cell lung cancer (NSCLC), pancreatic cancer and breast cancer [13,25,26,27,28]. The risk of DIILD is highest when used in combination with other agents, especially bleomycin, erlotinib and taxanes [25,26,28,29,30,31], with reported incidence rates of 1–20%. Mortality rates are generally low [26,27,28,32] except in severe cases requiring hospitalisation, where mortality reaches 20% [30]. In contrast to bleomycin, the dose relationship and timing of onset are less consistent [13,26,29].

A nationwide retrospective database study in Japan identified 428 cases of DIILD in 25,924 gemcitabine-treated patients [30]. The median time of onset was 65 days and the cumulative incidence was 1.1%, 1.5% and 1.9% at 3, 6 and 12 months. The crude incidence rates were similar after monotherapy (1.7%) and combination therapy (1.6%).

##### Epidermal Growth Factor Receptor (EGFR)-Targeted Agents

EGFR-targeted agents include small molecule receptor tyrosine kinase inhibitors (RTKIs) and monoclonal antibodies licenced for treatment of NSCLC, breast cancer and colorectal cancer [33,34]. The reported incidence of DIILD for the EGFR-RTKIs gefitinib and erlotinib is 1.2–1.6%, with an associated mortality rate of 22.8% [35,36]. DIILD following EGFR-RTKIs appears to be an early event, with studies of gefitinib and erlotinib reporting the highest incidence within 4 weeks of starting treatment [34,37].

In Japanese post-marketing surveillance studies, the incidence of DIILD with EGFR-directed monoclonal antibodies, such as panitumumab and cetuximab, was 1.3% and 1.2%, respectively, with a broad time to onset (median 101 days, range 17–431) [33,38]. Another study reported a median onset of 113 days (range 1–559) following the first dose of panitumumab, with 11/39 cases occurring after 6 months of therapy [39]. Notably, many patients in this series also received treatment in combination with other agents associated with DIILD risk. A single study in Japan reported mortality rates of 41.6% and 51.3% for cetuximab- and panitumumab-related DIILD, respectively [33].

##### Mechanistic Target of Rapamycin Protein (MTOR) Inhibitors

MTOR inhibitors are used predominantly to treat renal cell cancers and neuroendocrine tumours, and as anti-rejection agents in solid organ transplantation [40,41,42,43,44,45]. Sirolimus, temsirolimus and everolimus have all been associated with pulmonary toxicity [45,46,47]. A meta-analysis of 2233 everolimus-treated cancer patients in five clinical trials reported a DIILD incidence of 10.4% (all grades) and 2.4% (grade 3–4). Mortality data were not reported, and no associations with treatment duration, gender or cancer outcomes were observed [46]. Cases were observed in not only Japanese centres but also Western countries.

Post hoc analysis of computerised tomography (CT) data from clinical trials of temsirolimus and everolimus found a significantly higher incidence of radiographic changes consistent with DIILD (everolimus radiographic 53.9% vs. clinical 13.5%; temsirolimus 29% vs. 6%) [42,45,47].

In organ transplant recipients, variable incidence rates ranging from 2.8% to 12.7% have been reported in observational studies [41,48,49].

##### Immune Checkpoint Inhibitors

Checkpoint inhibitors of programmed cell death 1 (PD-1) and its ligands (PD-L1 and PD-L2) and cytotoxic lymphocyte antigen protein 4 (CTLA-4) are an emerging class of agents currently licensed in metastatic melanoma, NSCLC and Hodgkin’s lymphoma [50,51,52]. Immune-mediated reactions are well recognised [50]. A meta-analysis of clinical trials of PD-1 and PD-L1 inhibitors highlighted a DIILD incidence rate of 3.6% for PD-1 inhibitors (nivolumab, pembrolizumab) and 1.1% for PD-L1 inhibitors (avelumab and durvalumab) [52,53]. The incidence rate, severity and mortality of DIILD were all higher for PD-1 inhibitors compared with PD-L1 inhibitors, with a DIILD mortality rate of 8%. No association with dose or duration of treatment was observed [52].

Another observational study of 1826 cancer patients treated with checkpoint inhibitors reported a DIILD incidence rate of 3.5% [51], and a mortality rate of 9.4% for DIILD cases, which is similar to clinical trial data. Time to onset ranged from 0.2 to 27.4 months, with 42% occurring within 2 months of starting treatment. When used in combination therapy, the rates of DIILD were increased compared to single-agent use [54].

#### 3.2.2. Rheumatological Therapy

In rheumatology, analyses of DIILD are hampered by a background prevalence of ILD, especially in rheumatoid arthritis (RA). Furthermore, many DMARDs are immunosuppressive and associated with an increased risk of opportunistic infection, providing challenges in the differential diagnosis of worsening respiratory symptoms.

##### Methotrexate (MTX)

MTX is a mainstay agent in rheumatology and for the treatment of lymphomas and sarcomas. The incidence of DIILD in RA patients receiving low-dose MTX has been reported as 0.3–2.1% [55,56] Two meta-analyses compared rates of DIILD following MTX to other DMARDS in RA and non-RA inflammatory diseases [55,57]. In RA, the DIILD rate with MTX exposure was 0.28% (13/4544) compared to 0/4040 for other DMARDs (relative risk (95% CI) = 7.81 (1.76–34.72)) [55]. In the non-RA population, no increased risk was seen in MTX-treated patients [57]. Interestingly, no events were reported after 2002 in the RA meta-analysis, suggesting potential reporting bias or historic over-estimation of risk [55].

MTX-induced DIILD has a variable time and rate of onset and is not apparently dose-dependent [56,58,59]. In one study, 48% of cases developed within 32 weeks of treatment initiation [60]. Kremer et al. noted a mean time to onset of 23 days (range 3–112) [61]. Others, however, noted cases occurring up to 4 years after starting treatment, or after treatment cessation [60]. DIILD has also been reported to recur in approximately one-third of re-challenged cases [60,61] and carries a high mortality (10–30%) [59,60,61].

##### Leflunomide

Most reported data are from Japan. In one post-marketing surveillance study of 5045 Japanese patients taking leflunomide, new ILD occurred in 1.2%, and pre-existing ILD deteriorated in 5.7% of cases [62]. Most patients presented within 20 weeks of treatment initiation in one study [63]. Leflunomide-related mortality was 19% and 41% in two studies [63,64]. In a nested case-control study, Suissa et al. noted significant channelling bias which may explain some of the increased risk with leflunomide [65]. However, use of a loading dose and low body weight were significantly associated with DIILD, suggesting leflunomide toxicity [62,65]. Pre-existing ILD, smoking and prior MTX use have also been reported to increase DIILD rates for leflunomide [62,65]. Following a medical alert advising against drug loading and caution in patients with low body weight or pre-existing ILD, the incidence of ILD was reported to have reduced from 1.46% to 0.63% [62]. Conway et al. found no increased rate of adverse pulmonary reactions from leflunomide in a meta-analysis of clinical trials [66].

##### Biological DMARDs

Numerous cases of suspected DIILD associated with anti-tumour necrosis factor (TNF) agents have been published, although definitive evidence of causation remains controversial [67]. Post-marketing surveillance data from Japan reported an incidence rate of 0.6% for new or progressive ILD in patients treated with anti-TNF therapy [68]. This study did not have a control arm. Cohort studies have not demonstrated a difference in rates of incident ILD between patients treated with anti-TNF agents and other types of DMARD, but there are no observational studies which compare rates of DIILD [69,70]. In a review of published case reports, 15/52 (29%) patients with ‘DIILD’ on anti-TNF therapy died during follow-up, with 70% of deaths occurring within 5 weeks of symptom onset [71]. Mortality was highest in older patients, those with pre-existing ILD or those receiving concomitant immunosuppression. Two systematic reviews highlighted cases of potential DIILD associated with other biologic DMARDs, including tocilizumab (an interleukin 6 inhibitor) and rituximab [67,72]. Three systematic reviews included published cases of rituximab-induced ILD ranging from 7 to 45 cases [72,73,74]. The majority of cases were oncology patients presenting with acute or subacute ILD around the fourth cycle of treatment. Case fatality ranged between 18% and 37.5% [72,73,74].

#### 3.2.3. Other Drug Classes

##### Antibiotics

Nitrofurantoin is commonly used for the treatment and prophylaxis of urinary tract infections. DIILD accounts for 16–48% of nitrofurantoin-related adverse events reported in registry studies [75,76]. In a Swedish registry study of 447 nitrofurantoin-related DIILD, almost 90% were acute reactions [76]. The hospitalisation rate was 75% and mortality rates were 0.5% and 8%, respectively, for patients with acute lung reactions and chronic interstitial pneumonia [76].

An acute pulmonary reaction can occur within days of initiation, or within hours if there has been previous nitrofurantoin exposure [77]. The underlying mechanism is believed to be an acute hypersensitivity reaction, and most cases resolve quickly [77]. Chronic interstitial pneumonia is a rarer presentation mimicking pulmonary fibrosis [75,76,77], and is more common in patients on long-term prophylaxis [76]. Santos et al. performed a case-control study comparing DIILD with nitrofurantoin use compared to other antibiotics [78]. Overall, the relative risk (RR) of DIILD was not increased for nitrofurantoin. The absolute risk was higher for chronic compared to acute nitrofurantoin therapy (RR 1.53 chronic vs. acute use, *p* < 0.05), and for older patients (age >85 relative risk 1.99 for age 85 vs. <85, *p* < 0.05).

Regarding other antibiotics, daptomycin—an antibiotic usually reserved for life-threatening Gram-positive bacteria—has been associated with a risk of eosinophilic DIILD. In a retrospective study, 3/102 daptomycin-treated patients developed DIILD with eosinophilia [78]. A review of the Food and Drug Administration (FDA) pharmacovigilance database identified 7 definite, 23 probable and 38 possible cases of daptomycin-induced eosinophilic pneumonias between 2004 and 2010 [79]. All patients in this series recovered. 

##### Amiodarone

Amiodarone is one of the most common causes of DIILD in registries [10], with a reported incidence of 1.2–8.8% [80,81,82,83,84] and mortality of 3–37% [80,81,82,83,84]. A retrospective study of 500 patients treated with amiodarone in Japan identified 40 patients (8%) with DIILD occurring during a mean follow-up of 48 months [85]. The cumulative incidence at 1, 3 and 5 years was 4.2%, 7.8% and 10.6%, respectively, with an estimated annual incidence of 2.1%. Patients most commonly present with subacute DIILD [86,87]; however, an acute, frequently fatal form can occur [80]. One study evaluating 90-day outcomes in patients hospitalised for amiodarone-associated DIILD reported a 37% mortality rate with a median time to death of 17 days. Symptomatic recovery in survivors occurred over a median of 36 months, with improvement in radiological features of alveolitis but a high rate of fibrosis (66%).

Cumulative dose is an important risk factor for amiodarone-related DIILD, and the combination of high doses over longer periods is more strongly associated with DIILD than dose or duration alone [88].

### 3.3. Risk Factors for the Development of DIILD

Risk factors for the development of DIILD vary according to the disease, drug and population being treated. Certain risk factors have featured prominently across drugs.

Age: Increased age has been identified as a significant risk factor for DIILD for treatment with bleomycin, gemcitabine, EGFR-targeted agents, leflunomide, MTX, amiodarone and nitrofurantoin [23,30,33,58,62,76,77,85,95,104,107]. For bleomycin, dose reductions are recommended together with weekly chest radiographs and close follow-up after completion of therapy to monitor for DIILD in patients >60 years old. In contrast, no age association has been found with MTOR inhibitors [42,52,62].

Pre-existing lung disease: Pre-existing ILD or Idiopathic Pumonary Fibrosis (IPF) is an independent risk factor for DIILD with a wide range of agents [13,34,45,67,89,91,95,108,109]. For example, in NSCLC patients, prior ILD was associated with a 3.19-fold increased risk of DIILD in Japan [34]. Increased DIILD risk has also been associated with pre-existing Chronic Obstructive Pulmonary Disease (COPD), bronchiectasis and asbestosis [34,95,109,110].

Smoking: Smokers are at increased risk of DIILD when treated with gemcitabine, EGFR-targeted agents and methotrexate [20,33,56,58,95,108,109,110,111,112].

Drug dose: A clear dose-dependent relationship is well recognised for bleomycin, amiodarone and nitrofurantoin and is described in the Common Causative Drugs section. However, findings are not consistent for other agents across studies [41,45].

Underlying disease characteristics: In oncology, poor performance status and advanced or metastatic stages of disease are independent risk factors for DIILD [30,33,107,110]. One Japanese post-marketing study observed a 3-fold higher risk of DIILD for patients with NSCLC treated with gemcitabine compared to other cancers treated with gemcitabine [30]. Whilst there may be confounding due to a higher incidence of pre-existing ILD in NSCLC, this difference was not observed in studies of other agents used for this indication [46,95]. In RA, methotrexate DIILD risk was increased in patients with high inflammatory markers, low albumin, extra-articular disease and high levels of disability [67,111].

Sex: Male sex has been reported as a risk factor for DIILD in some studies following treatment with EFGR inhibitors, pemetrexed, methotrexate and amiodarone [33,58,85,95].

Other therapies: For gemcitabine, prior chemotherapy carried a relative risk of DIILD of 1.45 [30,33]. Conversely, two studies (one in erlotinib-treated and one in immune checkpoint inhibitor-treated patients) found that re-treatment with the same drug or another drug in the same class actually carried a lower DIILD risk [34,52]. Prior thoracic radiotherapy also increased DIILD risk in lung cancer patients [34]. In RA, prior MTX exposure increased the risk of leflunomide-induced ILD [65], and prior DMARD therapy was independently associated with a higher risk of MTX-induced DIILD [58]. However, such studies may be confounded by such patients having more severe or progressive disease.

Other risk factors: Other potential risk factors for DIILD include genetic susceptibility, higher alcohol consumption, renal dysfunction and diabetes [26,34,58]. Certain (Cytochrome P450) CYP enzyme polymorphisms increase the risk for drugs metabolised by CYP enzymes, and certain (human leukocyte antigen) HLA allelic variants have been linked with DIILD following erlotinib–gemcitabine combination therapy [113,114]. Weiner et al. reported that patients switching to sirolimus at a later stage of anti-rejection treatment were at higher risk of DIILD, as were patients with impaired renal function [115]. Higher rates were also observed in Japanese patients, and this may be a combination of genetic susceptibility and variation in reporting, which has been observed between different countries [5].

### 3.4. Radiological Investigation of DIILD and Prevalent Radiopathological Patterns

Unilateral or, more commonly, bilateral pulmonary infiltrates on a chest radiograph may be the first indication of DIILD [116]; however, 25–75% of chest radiographs are normal in cases of clinically suspected DIILD [89,116,117].

CT has higher sensitivity for detecting ILD features and is the imaging modality of choice [45,118]. The main limitations are exposure to ionising radiation, an issue minimised with modern scanners and the use of iterative reconstruction. To date, CT assessments have been non-specific for DIILD, as the numerous patterns of interstitial change are commonly seen in other ILDs.

Formal studies assessing CT in DIILD are limited by inconsistent terminology, many having been conducted prior to the current ATS/ERS ILD classification [1]. Pathological terms such as chronic interstitial pneumonia (CIP) are common in older imaging studies [14,15,16], but they have limited utility due to relatively poor agreement between radiological and pathological findings [15,119]. Several studies have compared histopathological and imaging findings in DIILD [12,15,16,73,119,120]. A prospective study of 42 patients with DIILD undergoing transbronchial lung biopsy (TBLB) or bronchoalveolar lavage (BAL) reported an overall diagnostic agreement of 67% [15], while a retrospective analysis of patients with DIILD undergoing TBLB (*n* = 4) or open biopsy (*n* = 16) at a single centre reported a lower diagnostic agreement (45%) [119]. In contrast, CT features of diffuse alveolar damage (DAD) are highly congruous with histopathological features of DAD [1] and confer high mortality [33,39,80,90,94,95,121,122]. In general, the correlation imaging pattern of CT and pathology is suboptimal [119].

DIILD most commonly manifests as ground glass opacification (GGO) with or without consolidation [16,17,44,80] and has a basal, peripheral and bilateral distribution, often affecting multiple lobes [43,91,92]. Heterogeneity in reporting makes the true incidence of each pattern difficult to establish. Changes resembling organising pneumonia (OP) are most commonly reported, followed by non-specific interstitial pneumonia (NSIP) and hypersensitivity pneumonitis (HP)-like changes [12,13,14,15,33,34,39,43,73,90,91,92,93,94,122]. NSIP is reportedly more common in hospitalised patients [12] and in chemotherapy-induced DIILD [12,13]. Reticular changes and volume loss (termed CIP in older studies) occur less commonly [73]. In the modern classification, this is most closely aligned with fibrotic NSIP or usual interstitial pneumonia (UIP) [1]. In some cases, hilar lymphadenopathy and pleural effusions were found, often when associated with eosinophilia [123]. Appearances consistent with sarcoidosis have been reported in at least six cases following treatment with interferon-alpha [15]. Another pattern of ground glass opacities with interlobular septal thickening, termed “crazy paving”, is recognised in DIILD, but is neither sensitive (present in 12% of DIILD) nor specific, as it is common in the context of heart failure [123].

Qualitative CT features are not specific to DIILD, as other causes of these radiological patterns, such as atypical infections (particularly in immunosuppressed patients) or connective tissue disease-associated ILD, may confound interpretation [44,45]. In addition, the radiological patterns of DIILD for the same drug are highly variable (e.g., NSIP, DAD and COP patterns are seen in amiodarone-induced ILD [30]) and, conversely, the same pattern can be a feature of numerous drugs (e.g., OP-like pattern is seen in a number of agents, including EGFR RTKIs, checkpoint inhibitors and amiodarone [39,51,84]).

### 3.5. Non-Imaging Diagnostic Investigations

#### 3.5.1. Pulmonary Physiology

Pulmonary physiology is important in the assessment of suspected DIILD but, like CT, lacks specificity. Reduction in D_LCO_ is a presymptomatic feature of DIILD, and changes in FVC correlate with clinical progression in bleomycin-treated patients [18,124]. The sensitivity of pulmonary function tests (PFTs) varies within studies. One historic study in suspected bleomycin lung reported abnormal PFTs in only 31/150 patients [18]. Yamada et al. evaluated the diagnostic accuracy of percentage change in D_LCO_ in patients treated with amiodarone [85]. Sensitivities of 76%, 68% and 59% were found for a 10%, 15% and 20% reduction in D_LCO_, respectively. In a study of nitrofurantoin-induced DIILD, all patients had impaired D_LCO_ but only 2/17 had abnormal FVC [77].

#### 3.5.2. Bronchoalveolar Lavage (BAL)

BAL findings, including raised lymphocyte, neutrophil and eosinophil counts [122,125,126,127], are not specific for DIILD, as they also occur in types of inflammatory or infective lung disease [126]. Reversal of the CD4:CD8 lymphocyte ratio has been reported in some, but not all, studies, but again is not specific for DIILD [127,128,129,130]. Other reported findings include cellular abnormalities, such as nuclear enlargement and hyperchromasia, lipid inclusions and haemosiderin-laden macrophages [15,131]. The presence of reactive type II pneumocytes has also been described in severe cases of DIILD [16].

Opportunistic infection is high on the differential diagnosis of most DIILD. In some studies, positive BAL microbiology led to a revised diagnosis in suspected DIILD cases [41,111,116,132]. In a study of 26 everolimus-treated patients initially diagnosed with DIILD, 12 (46%) were subsequently diagnosed with *Pneumocystis jiroveci* [41]. Currently, the key role of BAL is to aid in the exclusion of other causes, especially infection.

#### 3.5.3. Lung Biopsy

The role of lung biopsy has been limited to small studies, and almost all histopathological patterns have been reported in DIILD; however, none are specific for DIILD [15,16]. There is, therefore, limited evidence for the routine use of biopsy in the diagnosis of DIILD but, like BAL, it may be useful in selected cases where there is diagnostic uncertainty or to exclude other causes.

#### 3.5.4. Circulating Biomarkers

Krebs von den Lungen-6 (KL-6) is a mucin-like glycoprotein secreted by type II alveolar pneumocytes and bronchial epithelial cells in response to damage and regeneration in the context of ILD. In a prospective study, increased KL-6 was observed in 53% of DIILD patients and correlated with the DAD pattern and more extensive lung involvement [14]. Changes in KL-6 over time corresponded with the clinical course. A second study reported a predictive association between the ratio of KL-6 to sialyl SSEA-1 (SLX) and subsequent DIILD in lung cancer patients undergoing chemotherapy [90]. The specificity of KL-6 in DIILD has not, however, been established. Other biomarkers, including peripheral eosinophilia and raised inflammatory markers, are non-specific and not diagnostic for DIILD.

### 3.6. The Role of Glucocorticoids (GCs) in the Treatment of DIILD

Our review was limited by a lack of randomised data, missing data on dose and duration, and variation in criteria for patient selection and dose. Assessing the impact of GC therapy on resolution of DIILD or survival was also hampered by the common practice of introducing steroids contemporaneously with the withdrawal of the offending drug. Table 3 summarises studies where either dosing information and/or outcomes from GC treatment were available.

#### 3.6.1. Efficacy

The reported efficacy of GC treatment in DIILD varied widely. In a series of 75 cancer patients with irinotecan-induced DIILD treated with GC, 46 (61%) recovered and 22 (29%) died [93]. In 10 pemetrexed DIILD patients treated with GC, five patients (50%) responded, four (40%) failed to respond and one died [95]. Rebattu et al. also reported 100% recovery after drug discontinuation and GC therapy in six DIILD cases associated with combination gemcitabine and docetaxel therapy [133]. In one series of all-cause DIILD, 62% (29/47 patients) received GCs and the remaining 18 patients recovered without supportive GC therapy [12]. The retrospective nature of these studies and the lack of specific criteria for the use of GC means there is a high risk of channelling bias, i.e., GCs tend to be used more commonly in those with severe disease and a DAD pattern.

#### 3.6.2. GC Dose and Duration

GC doses ranged widely and included high-dose oral and IV methylprednisolone regimes, with dosing and duration in part guided by the radiological pattern [59,134]. Takatani et al. reported a median cumulative dose of 5240 mg prednisolone for DAD, compared to 2722 mg for OP, 415 mg for HP and 264 mg for NSIP groups [122]. Weak supportive evidence for the role of GCs is suggested by an increased risk of DIILD relapse when GCs are stopped or tapered early (within 3 months of onset) [80,116]. The merit of high dosages is not established.

#### 3.6.3. DIILD Subsets and GC Responses

The DAD pattern has the poorest prognosis, and, in one series, no DAD patients improved with GC treatment, and the overall mortality was 37.5% [12]. Reported improvements with GC therapy for other radiological patterns were: 75% (3/4 patients) for OP, 45.8% (11/24 patients) for NSIP and 36.4% for HP pattern [12].

Although GC therapy was used in many studies, there is currently no evidence on which to base recommendations for GC use in DIILD. The use of GC in therapy is recommended in severely affected patients, with dosing regimens at the discretion of the attending physician. Further studies are required in order to develop more detailed treatment recommendations.

### 3.7. Prognosis

DIILD prognosis varies between drugs and different studies. Complete recovery is possible following dose reduction, drug withdrawal and/or concomitant GC use [17,34,45,73,91,110]. Nevertheless, a significant proportion fails to improve, or follows a progressive clinical course [29,34,91,110]. DIILD mortality is often due to respiratory failure, multiorgan failure, progression of the primary underlying disease or as an adverse effect of GC therapy (e.g., infection) [80,106]. In the context of oncology, mortality ranges from 14 to 51.3% [13,22,30,33,34,40,93,109], whilst in non-cancer settings, mortality ranges from 0 to 41% [26,59,60,61,63,64,71,106].

#### Factors Predicting Mortality

Clinical characteristics: Acute and severe presentations are the most consistent predictors of mortality. In particular, a requirement for mechanical ventilation is associated with mortality rates of >60% [135,136]. Rapid symptom onset, higher initial disease severity and hypoxaemia at presentation also predict mortality [63,64,80]. Pre-existing ILD, male sex, age >65 years and a diagnosis of NSCLC are also associated with higher case fatality rates [93]. In NSCLC patients, a poor performance status (2–4), ≥2 prior chemotherapy regimens and <50% remaining normal lung area also predict DIILD mortality [34,108].

Radiological patterns: For CT, a greater extent of lung injury and a homogenous pattern are associated with higher mortality in amiodarone-treated patients [106]. DAD and NSIP patterns also predict poorer outcomes [80,119] (with the DAD pattern, 40–83.3% mortality) [33,34,95]. Honeycombing with interstitial pneumonia is also associated with higher mortality [34]. Of note, however, is that full recovery has been seen with diffuse ground glass opacities [95], and some studies have not found CT patterns to be prognostic [119,135].

Other: For BAL, the presence of desquamated type II pneumocytes is associated with mortality [16]. Others have also found that circulating and/or BAL KL-6 and heat shock protein 47 are associated with higher mortality [12,119,121,122]. Their association with a more severe clinical presentation and with DAD, however, means that their incremental value as prognostic markers remains unclear.

## 4. Discussion

DIILD may affect a wide spectrum of patients globally, with a significant impact on survival in multiple contexts. Evidence on the true incidence and mortality, case definition, diagnostic tests and optimal treatment of DIILD is lacking. The TRISTAN Consortium has set out to address some of these gaps, and this systematic review provides a baseline assessment of what is already known about this condition and Table 4 highlights the key findings of this PRISMA compliant systematic review.

Few studies have examined the incidence rates in the general population, and the available pharmacovigilance studies likely significantly underestimate the risk. A significant risk of DIILD has been demonstrated with newer agents, such as EGFR-targeted therapies, highlighting the urgent need to investigate the true scale of DIILD and its impact on treatment and mortality in a contemporaneous population.

Whilst we identify a large number of relevant papers in this review, the majority of studies were of low quality, with a high risk of bias. The lack of standardised case definition used across studies made it impossible to pool data and conduct a formal meta-analysis. While a large number of studies reported rates of new ILD occurrence and pulmonary toxicity with specific agents, there was a lack of high-quality studies investigating DIILD in a non-agent-specific setting. Other significant factors limiting our ability to draw firm conclusions included the over-representation of some geographical areas, such as Japan, where rates of ILD and reporting methods may differ from other parts of the world. There was also significant confounding in a number of studies, and limitations in study design and sample size all added to difficulties in data synthesis.

From published data, there are no radiopathological patterns specific to DIILD and there are no investigations which, alone or in combination, can be confidently used to diagnose DIILD. Imaging with CT plays an important role in the identification of lung changes, and bronchoscopy is helpful for excluding infection. All tests, however, lack specificity in DIILD; therefore, the diagnosis largely remains one of exclusion. The lack of specific diagnostic markers for DIILD has an impact on drug development, particularly in cancer and rheumatology populations, which have a high background prevalence of ILD and significant respiratory infection risk. Better biomarkers to detect early safety signals and to distinguish between disease and drug-related ILD are needed. More specific imaging biomarkers derived from quantitative CT analyses, positron emission tomography or MRI (e.g., hyperpolarised 129Xe MRI, oxygen-enhanced MRI, dynamic contrast-enhanced MRI) better characterise lung structure and function, and may facilitate the development of biomarkers specific to DIILD [137,138,139].

Evidence for managing DIILD was distinctly lacking. In some studies, agents were continued even with grade 3 DIILD [45]. The risk of development and progression of DIILD must always be balanced against the negative impact of stopping the drug on outcomes/survival, and this delicate balance may vary depending on the condition and treatment efficacy. The literature had no consistent approach to decision making on drug withdrawal and no robust evidence base for the use of GCs. We found a number of possible risk factors for the development of DIILD and prognosis, which could be considered when developing a consensus on the management approach. We have identified points to consider in any approach to GC use in DIILD. However, prospective studies are required, with more detailed recommendations needed to aid decision making on drug initiation, withdrawal and monitoring in high-risk groups.

It is important to note that this review does not provide a comprehensive catalogue of all drugs implicated in the causation of drug-induced lung disease, specifically as we excluded case reports and studies with a sample size of less than 10. The most recent data from the comprehensive website Pneumotox (www.pneumotox.com) highlight 1406 drugs, substances or procedures reported as having caused pulmonary injury (personal communication Ph Camus through his website, 3 October 2018). Approximately 800 of these agents/procedures had less than 10 reports on the website. While not all of these substances are licensed drugs nor cause an interstitial lung disease pattern of injury, this underlines a limitation of the current review.

Overall, this systematic review informs the formulation of a research agenda in DIILD, and we propose several key areas for further investigation and development, including:

(i) A standardised case definition for the study of DIILD to be used in clinical trials and observational studies.
(ii) Validation of better biomarkers for detecting early DIILD and discriminating from other causes of ILD.
(iii) An evidence base for the management of DIILD, including through clinical trials to the efficacy and optimal dosing of GCs in DIILD.

The TRISTAN Consortium will address this research agenda and will focus on prospective studies using novel imaging methods and biomarkers, with accompanying pre-clinical studies to interrogate mechanisms and validate imaging methods. Our aim is to develop better biomarkers for use, both in drug development and clinical practice, for the diagnosis and monitoring of DIILD and other ILD subtypes. We also aim to undertake prospective work to estimate the burden of DIILD and take steps towards developing a consensus on management. 

## Figures and Tables

**Table 1:** Grading of drug-induced interstitial lung disease (DIILD) based on the National Cancer Institute Common Terminology Criteria for Adverse Events [2].

Grade 1 (mild)	Asymptomatic, radiographic findings only
Grade 2 (moderate)	Symptomatic, not interfering with activities of daily living
Grade 3 (severe)	Symptomatic, interfering with activities of daily living or oxygen indicated
Grade 4 (life-threatening or disabling)	Life-threatening, or ventilator support required
Grade 5 (fatal)	

**Table 2:** Summary of specific classes or agents associated with DIILD identified from literature review of lung disease.

Drug/Class	Number of Studies	Quality	Study Design	Patient Population	Sample Size (Range)	Case Definition of DIILD	Estimated Incidence (Range)	Estimated Mortality in Those with DIILD (Range)
Cancer Therapies								
Bleomycin [18,19,20,21,22,23,24]	7	Moderate = 3 Low = 3 Very low = 1	Meta-analysis = 2 Observational studies = 5	Various cancers (1 meta-analysis in ovarian sex cord stromal tumours and 1 in all cancer RCT data)	22–1147	variable	Meta-analyses: 6.8–15% Other studies: 6.8–21%	Meta-analyses: 8.1–23% Other studies: 0–48%
Gemcitabine [13,25,26,27,28,29,30,31,32]	9	Moderate = 2 Low = 6 Very low = 1	Meta-analysis = 2 Clinical trial = 3 Observational = 4	Cancer (predominantly pancreatic and non-small cell lung cancer but also others)	Meta-analysis: 1308–1742 Others: 26–2440	variable	1.1–3.9%	0–22%
Epidermal growth factor receptor-targeted therapies (EGFR)								
Erlotinib [34,35,36,89,90]	5	Moderate = 2 Low = 3	Meta-analysis = 2 Post marketing surveillance = 2 Observational = 1	Non-small cell lung cancer	341–9909	variable	0.9–5.9%	31–45%
Gefitinib [34,35,36,37]	4	Moderate = 2 Low = 2	Meta-analysis = 2 Post marketing surveillance = 2	Non-small cell lung, breast and colorectal cancer	70–5468	variable	1.9–3.5%	18–44%
Panitumumab [33,39]	2 (but reporting from same cohort)	Moderate = 2	Post marketing surveillance	Colorectal cancer	3085	Expert case review	1.3%	51.3%
Cetuximab [38]	1	Moderate = 3	Post marketing surveillance	Colorectal cancer	2006	Physician reported	1.2%	41.6%
Mechanistic target of rapamycin protein (MTOR) inhibitors								
Everolimus [40,41,42,43,45,46,48,49]	8	Moderate = 3 Low = 3 Very low = 2	Meta-analysis = 1 Clinical trial = 2 (same trial 2 separate published analyses) Observational = 5	Neuroendocrine cancer Renal cell cancer Renal transplant	40–2261	Variable, including radiographic signs of DIILD	2.8–58%	5.4–20%
Temsirolimus [44,47]	2	Low = 2	Meta-analysis = 1 Clinical trial = 1 Observational study = 1	Neuroendocrine cancer Endometrial cancer Renal cell cancer	22–408	Variable	29–36%	n/a
Sirolimus [48]	1	Very low = 1	Observational	Renal/pancreas transplant	115	Physician reported	9.5%	0%
Check point inhibitors (CPI)								
All CPIs [51,52,53]	3	High = 2 Moderate = 1	Meta-analysis = 2 Observational = 1	Non-small cell lung cancer	1826–3232	variable	1.1–3.6%	8–9.4%
Ipilimumab [91]		Low = 1	Observational = 1	Melanoma	146	Radiographic evidence of DIILD	5.44%	n/a
Nivolumab [92]	1	Low = 1	Post hoc pooled clinical trial analysis = 1	Cancer (various types)	170	Physician reported events	11.7%	0%
Other agents identified								
Irinotecan [93]	1	Low = 1	Post marketing surveillance	Cancer (various types)	8864	Physician reported	0.74%	24%
Rituximab [67,72,73,74]	4	Very low = 4	Systematic reviews = 3 Case series = 1	Predominantly cancer but other indications included	16–52	Variable	n/a	n/a
Imatinib [94]	1	Low = 1	Post marketing surveillance	Leukaemia	6	Physician reported	n/a	6/6 resolved
Pemetrexed [95]	1	Moderate = 1	Post marketing surveillance	Mesothelioma Non-small cell lung cancer	903	Expert committee review	1.8%	
Granulocyte colony stimulating factor [96]	1	Low = 1	Observational	In conjunction with chemotherapy	40 treated vs. 25 with chemotherapy along	Physician reported	0.2% vs. 0% in the control group	n/a
Rheumatology drugs								
Methotrexate [55,56,57,58,59,60,61,67]	8	Moderate = 3 Low = 4 Very low = 1	Meta-analysis = 2 Clinical trial = 3 Observational = 2 Case series = 1	Rheumatoid arthritis Psoriasis, psoriatic arthritis or inflammatory bowel Primary biliary cirrhosis	29–3188	variable	0.06–15%	10–33%
Tumour necrosis factor inhibitors [67,68,69,70,71,72,97,98]	8	Moderate = 4 Low = 1 Very low = 3	Post marketing surveillance = 3 (2 papers report on 1 study) Observational study = 3 Systematic review of case reports = 3	Predominantly rheumatoid arthritis but cases in other diseases	233–13,894	variable	0.6%	32%
Leflunomide [62,63,64,65,66]	5	Moderate = 1 Low = 3 Very low = 1	Meta-analysis of RCTs = 1 Case control via claims database = 1 Post marketing surveillance = 2 Case series = 1	Rheumatoid arthritis	2274–62,734	variable	0–1.2%	19–41%
Cardiology drugs								
Amiodarone [80,81,82,83,84,85,86,87,88,99,100,101]	12	Moderate = 2 Low = 5 Very low = 5	Observational = 7 Case series = 5	Cardiovascular disease	13–500	Variable, often not restricted to DIILD	1.2–8.8%	0–41%
Bepridil [102]	1	Low = 1	Observational	Cardiovascular disease	222	Standardised definition	6.3%	0%
Statins [103]	1	Very low = 1	Observational (Adverse events reporting database)	Cardiovascular disease/prevention			1/40 adverse event reports for statins were ILD	n/a
Anti-infection agents								
Nitrofurantoin [75,76,77,78,104]	5	Low = 3 Very low = 2	Case-control study = 1 Registry study = 1 Post marketing surveillance = 1 Case series = 2	Chronic and acute treatment of urinary tract infection	10–70,804	Variable, some used “any ILD” after use of drug	3.65%	1.34%
Daptomycin [79,105]	2	Low = 2	Observational study = 1 Post marketing surveillance = 1	Infection (one study specifically infective endocarditis)	58–102	Variable	2.9%	n/a
Interferon [106]	1	Very low = 1	Systematic review of case reports	Hepatitis C	25	Variable	n/a	n/a

**Table 3:** Summary of studies which included information on use of glucocorticoids.

Author	Drug	Patient Population	Sample Size	Glucocorticoids Dose (Oral or IV)	Response
Mankikian et al. [80]	Amiodarone	DIILD	46	Median dose of 1 mg/kg 15 surviving patients followed and 9 (60%) received glucocorticoids for 3–29 months. All surviving patients successfully had glucocorticoids withdrawn	76% got glucocorticoids but no obvious difference in survival outcomes. Three patients treated for <3 months relapsed and glucocorticoids restarted. No relapse in patients treated for >6 months
Kakugawa et al. [12]	Various	DIILD	47	29 of 47 patients received glucocorticoid therapy. Decision on glucocorticoid therapy was physician-based rather than protocol-based. No dosing information available	None of the patients with a DAD pattern on HRCT improved with glucocorticoid treatment, and DAD group had a 37.5% mortality. 75% of those with OP pattern on HRCT (3 of 4) improved with glucocorticoid treatment. With an NSIP pattern, 45.8% (11 of 24 patients) improved with glucocorticoid treatment. Hypersensitivity pneumonitis (HP) pattern was associated with a 36.4% response to glucocorticoid therapy.
Ki et al. [134]	Bleomycin with cisplatin and vincristine	Cervical cancer patients treated with prior mentioned agents [59]	61 (7 cases of DIILD)	4 with bleomycin injury received glucocorticoid Different regimens within the study. 1 patient who improved received 40 mg/day methylprednisolone, followed by 10 mg daily. 2 acutely ill patients received IV methylprednisolone 500 mg/day × 3 days. 1 patient received 1 mg/kg/day prednisolone, then 0.5 mg/kg	Of these 4 patients, 2 died, 1 improved, 1 non-responder. Insulin-dependent diabetes developed in 2 patients
Kim et al. [105]	Daptomycin	Suspected DIILD	58 (7 definite DIILD cases, 13 probable cases)	No dosing information Definite cases: 5 of 7 received glucocorticoid (1 intravenous) Probable cases: 9 of 13 received glucocorticoid	No deaths 1 required long-term treatment
Rebattu et al. [133]	Gemcitabine with docetaxel	NSCLC patients treated with prior mentioned agents	49 (6 DIILD cases)	6/6 received glucocorticoids	All recovered
Ohnishi et al. [94]	Imatinib	DIILD	27	19/27 received high dose glucocorticoids 5/27 moderate dose glucocorticoids3/27 no treatment	7/27 resolved 16/27 improved 4/27 no improvement
Sharma et al. [59]	Methotrexate	Primary biliary cirrhosis patients treated with methotrexate	43 (6 DIILD cases)	5/6 received prednisolone 60 mg IV daily Duration of intravenous route and glucocorticoids taper unclear	4/5 given glucocorticoids responded, 1 patient died from liver decompensation
White et al. [45]	Everolimus	Advanced renal cell cancer patients treated with everolimus	416 (37 DIILD cases)	16/37 patients received glucocorticoids All 10 patients with grade 3 pneumonitis received glucocorticoids	10 patients with grade 3 pneumonitis who received glucocorticoids 3/10 continued everolimus: 1 died and 2 recovered 7/10 discontinued: 5 recovered, 1 had ongoing disease, 1 died
Tomii et al. [95]	Pemetrexed	Mesothelioma and NSCLC DIILD patients	1586 (10 DIILD cases)	10 cases, all of which received glucocorticoids	5/10 patients deemed glucocorticoids responsive, 1 indeterminate, 4 non-glucocorticoids responders died
Osawa et al. [33]	Panitumumab	Colorectal cancer patients treated with panitumumab	3085 (39 DIILD cases)	No dosing information available	Minimal information on glucocorticoid impact other than statement that most of the 20 patients who died had received glucocorticoids
Yoshii et al. [93]	Irinotecan	Cancer patients treated with irinotecan	8864 (153 DIILD cases, 83 with clinical information)	75/83 patients received glucocorticoids No dosing information available	46/75 of those treated recovered or improved, 5/75 no response, 22/75 died, 2/75 unknown outcome DAD pattern associated with lack of response to glucocorticoids
Liote et al. [73]	Rituximab	DIILD	45	27/45 cases of rituximab DIILD received glucocorticoid. Dosing unclear. Some patients received 1 mg/kg of body weight concomitantly with re-challenge.	No recurrence of rituximab injury in 3 patients receiving re-challenge with rituximab and concomitant 1 mg/kg methylprednisolone Early onset acute presentation: 5 patients all received glucocorticoids, 2 died Late onset chronic presentation in 3 patients who recovered with glucocorticoid therapy Authors recommend longer period of glucocorticoids usage rather than just boluses at each rituximab infusion, and a gradual taper to avoid rebound
Takatani et al. [122]	Various	DIILD		DAD group received median cumulative glucocorticoids dose of 5240 mg, range 1000–9195 mg; NSIP group median of 264, range 0–735 mg; HP group median 415, range 0–4470 mg; OP group median 2722, range 0–7835 mg	Days of oxygen therapy correlated well with cumulative doses of glucocorticoid therapy, i.e., the sicker patients received more glucocorticoids. OP pattern patients showed full recovery with glucocorticoids. No deaths in this group of 34 non-chemotherapy DIILD pts. 11 pts recovered fully without glucocorticoids
Chap et al. [116]	Cyclophosphamide, cisplatin and BCNU	Breast cancer patients treated with prior mentioned	64 (37 cases of DIILD)	37/37 treated with prednisolone 60 mg oral twice daily × 10 days, then 30 mg/day × 1 week, 20 mg/day × 1 week, 15 mg/day × 1 week, followed by 5 mg taper on daily dose each week. Initiation of prednisolone based on scoring system; crackles on lung auscultation = 2, drop in D_LCO_ by >10% from baseline = 3, drop in O_2_ saturation ≥4% with 2 min walk = 3, interstitial infiltrates on CXR = 3. Patients with a score ≥6 received prednisolone as above.	Glucocorticoid therapy associated with rapid clinical improvement in “most patients” (absolute numbers not available). 11 patients required prolonged prednisolone therapy (4–8 months), having experienced exacerbation of symptoms when prednisolone reduced to 15–20 mg/day
Hamada et al. [30]	Gemcitabine	pancreatic, lung, urothelial, breast, ovarian	25,924 (428 cases of ILD not verified as DIILD)	363/428 (84%) patients with ILD received either oral or intravenous glucocorticoids	20% of hospitalised DIILD patients with severe disease died, no data on glucocorticoid-treated group outcome versus non-glucocorticoid-treated patients

Abbreviations: DILD = Drug induced Interstitial Lung Disease; DAD = Diffuse alveolar damage; HRCT = High resolution Computer Tomography; OP = Organising Pneumonia; NSIP = Non specific interstitial pneumonia. And HP = Hypersensitivity pneumonitis.

**Table 4:** Key findings for each sub-question.

What is the incidence and prevalence of DIILD?
Incidence rates estimated between 0.41 and 12.4 per million per annum DIILD accounts for 3–5% of prevalent cases of ILD
What drugs are commonly associated with DIILD?
Cancer drugs followed by rheumatology drugs, amiodarone and antibiotics are the most common causes of DIILD Risks are highest when causative agents are used in combination Some, but not all, drugs are associated with a dose-dependent risk of DIILD Presentations and outcomes can vary even with the same agent
What are the risk factors for developing DIILD?
Smoking and pre-existing lung disease are significant risk factors for many agents Other risk factors for some, but not all, drugs are increasing patient age, drug dose, male gender, prior therapy, high alcohol intake, presence of comorbid conditions and genetic susceptibility factors
Radiological investigation of DIILD and the prevalent radiopathological patterns
Plain chest X-ray is often normal at presentation in DIILD CT is the imaging modality of choice in DIILD CT alone cannot discriminate between DIILD and other types of ILD Different radiopathological patterns of ILD can occur with the same causative agent No characteristic radiopathological findings are characteristic or pathognomic of DIILD, but OP, followed by NSIP and HP, are the most frequently seen patterns
What is the role of non-imaging diagnostic investigations?
Lung biopsy is not routinely indicated for investigation of DIILD BAL is an important investigation for the exclusion of infection There are currently no validated circulating biomarkers for the diagnosis or prognosis of DIILD
What is the impact of glucocorticoid (GC) therapy on DIILD outcome?
There are no robust or comparative studies evaluating the adjunctive role of GC therapy alongside withdrawal of the causative drug There is low-quality evidence to support the efficacy and dosing of corticosteroids by grade of severity and radiopathological subtype of DIILD A pragmatic approach to use of GC is warranted, but further prospective studies are required to investigate further
What if any factors predict prognosis?
Prognosis is highly variable between agents and patient populations DAD pattern of DIILD is associated with high mortality, but CT pattern alone is not consistently found to be a predictor of mortality Severity at presentation and acute onset are the most consistent predictors of mortality
